# Overexpression of MMP-3 and uPA with Diminished PAI-1 Related to Metastasis in Ductal Breast Cancer Patients Attending a Public Hospital in Mexico City

**DOI:** 10.1155/2016/8519648

**Published:** 2016-11-15

**Authors:** Luis Miguel Barajas-Castañeda, Evelin Cortés-Gutiérrez, Francisco Mario García-Rodríguez, Rafael Campos-Rodríguez, Eleazar Lara-Padilla, Fernando Enríquez-Rincón, María Eugenia Castro-Mussot, Paula Figueroa-Arredondo

**Affiliations:** ^1^Departamento de Inmunología, Escuela Nacional de Ciencias Biológicas, Instituto Politécnico Nacional, Prolongación de Carpio y Plan de Ayala S/N, 11340 Mexico City, Mexico; ^2^Servicio de Anatomía Patológica, Hospital Juárez de México, Secretaría de Salud, Av. Instituto Politécnico Nacional 5160, 07760 Mexico City, Mexico; ^3^Unidad de Oncología, Hospital Juárez de México, Secretaría de Salud, Av. Instituto Politécnico Nacional 5160, 07760 Mexico City, Mexico; ^4^Departamento de Investigación, Escuela Superior de Medicina, Instituto Politécnico Nacional, Plan de San Luis y Salvador Díaz Mirón S/N, 11340 Mexico City, Mexico; ^5^Laboratorio Nacional de Servicios Experimentales (LaNSE), Centro de Investigación y Estudios Avanzados del Instituto Politécnico Nacional, Av. Instituto Politécnico Nacional 2508, 07360 Mexico City, Mexico

## Abstract

Extracellular matrix metalloproteases and the fibrinolytic system are important protease systems interacting with each other in charge of remodeling and recycling of tissues. Their role in tumor invasion and metastasis is often discussed. In this study several metalloproteases such as MMP-1, MMP-3, MMP-9, and TIMP-1 together with molecules from the fibrinolytic system like uPA, its receptor uPAR, and its inhibitor, PAI-1, were studied by immune-histochemistry to establish a comparison with and without metastasis. From the (118) primary tumors of Mexican patients with ductal breast cancer studied, 56% were grade II and 69% were size T2; the group with metastatic ganglia included 64 samples (54.3%). In patients with metastasis the estimated expression of MMP-3 and uPA (resp., 28% and 45%) was higher than that from no metastatic tumors; it means there is higher expression of both markers in metastatic tumors (*p* < 0.05). At the same time, metastatic tumors showed statistically significant lower signal of PAI-1 (24%) than tumors without metastasis (*p* < 0.05). We concluded that overexpression of MMP-3 and uPA, altogether with diminished expression of PAI-1 from metastatic tumors, might be a crucial step towards metastasis in ductal breast cancer. Nevertheless, additional studies in different populations are necessary to establish a pattern.

## 1. Introduction

Breast cancer is the most common kind of women cancer worldwide, and the main cause of death due to neoplasia in underdeveloped countries such as Mexico [1]. One of the main problems of this cancer is delayed detection, increasing the risk of metastasis [2]. In breast cancer the lymphatic axillar ganglia are the starting point and most easily accessed site of metastasis and this event is almost the only prognostic sign of metastasis towards distant locations such as lungs, liver, bones, and brain. Distant location metastasis is the main cause of death [3–5] for this kind of cancer patients. An essential process in metastasis development is the processing or recycling of extracellular matrix (ECM) by the migrant tumor cells. This processing allows the tumor cells to invade surrounding tissues, be able to accessing blood vessels, and finally start building metastatic* de novo* formations in different tissues and organs. In tumor cells this processing is influenced mainly by the activity of proteases secreted by they themselves, but proteases can also come from other kinds of resident cells in the microenvironment [6–9].

Several kinds of proteases are actively processing extracellular matrix, among them there are the matrix metalloproteinases (MMPs) and serine-proteinases from the fibrinolytic system [10]. Under physiologic conditions, for example, tissue remodeling, ovulation, and wound healing, a very precise feedback regulation by proteolytic degradation machineries takes place. In cancer nevertheless, this fine equilibrium is disrupted probably facilitating metastasis [11–13], and from there our interest in establishing alterations on these systems in metastatic tumors of Mexican patients. Information of the molecules, appearing early or disrupting their behaviors in metastasis, may help predict metastasis from biopsies before surgery, or perhaps they could be used as indicators to select patients for immediate intervention, or perhaps even become suitable targets for medical treatment.

The MMPs system comes to mind first because it is effectively active; it is composed of a family of Ca^2+^ and Zn^+^ dependent-endopeptidases, processing different components of ECM. There are 26 MMPs already described, sharing biochemical structure and functions; however they differ in their substrate specificity [14, 15].

In the other hand, the fibrinolytic system is constituted mostly by plasmin activators such as uPA (urokinase-type plasminogen activator). This protein is important for coagulation, it specifically converts inactive plasminogen to active plasmin, but also plasmin is an important protein for remodeling since it is the enzyme responsible of degrading a variety of ECM proteins, including preprocessed metalloproteinases such as MMP-1, MMP-3, MMP-7, and MMP-9 [16–18]. These proteins are already regarded as important molecules in tumor invasion and metastasis; therefore we wanted to search for them in breast cancer tumors with and without metastasis.

In previous studies researchers reported significantly higher global expression of MMP-1, MMP-7, and MMP-14, TIMP-1 and TIMP-3 in the core of breast cancer tumors, in comparison with lymphatic axillary ganglia, but expression of the same markers in ganglia did not appear associated with prognosis of development not in near, neither in distant metastasis [19]. Besides, primary tumors and metastatic ganglia showed correlation with elevated uPA, whereas PAI-1 did not show correlation with any of them [20].

Nevertheless, the precise mechanism that MMPs and fibrinolytic system proteases use to participate in the metastatic process of breast cancer is still imprecise, particularly events taking place in lymphatic ganglia. The purpose of our study was to evaluate MMPs and fibrinolytic system components in tumor samples, derived from Mexican patients with ductal breast cancer, attending the Hospital Juárez de México with and without metastasis in ganglia and hopefully be able to distinguish abnormalities. The study was performed in order to observe, estimate, and compare the relative expression of these molecules by immunohistochemistry procedures and optical microscopy, followed by examination of signal intensities with a data processing software. The derived knowledge may perhaps help improve our ability to indirectly evaluate the performance of those feedback regulated systems, in the tumors with and without metastasis. Then, the comparison with the metastasis of primary lymphatic ganglia was also achieved.

## 2. Materials and Methods

### 2.1. Experimental Design

The present study was designed to detect differences in ductal breast cancer tumors with metastasis, from those with no metastasis; for that reason, two main groups of samples were collected: N0 were tumors without metastasis and N1 tumors with one or more metastatic ganglia. Once the selected molecules were identified in the tumors by immunohistochemistry procedures, the image analyzed by a suitable computer software, and their signals processed to establish statistically significant differences, those molecules showing differential expression were also tested in the metastatic ganglia for further characterization. Although from this approach there is no need of a group of healthy ductal breast tissue samples, such healthy samples were included for standardization of immunohistochemistry procedures. Also breast cancer tumors other than ductal were tested, as tissue controls (results not shown).

### 2.2. Biologic Materials

Since this is a retrospective study, all samples analyzed here were collected as part of routine diagnoses and treatment; therefore patients were not required to sign a consent information letter other than the one they signed to allow procedures related to their treatments. Ductal breast cancer biopsies of women from different areas of the country were selected due to their availability in the pathology collection.

The samples used here were taken during surgery procedures performed in 2008–2012, from women attending the Oncology service in the Hospital Juárez de México located in Mexico City.

This retrospective study included 118 paraffin embedded biopsy samples, diagnosed by histopathology technique using the classical hematoxylin-eosin (H&E) composed dye.

Most of the patients underwent total mastectomy and lymphatic node dissection (total for those with primary breast cancer). Average age of the patients was 51.6 (among 30 to 80 years old). Patients were selected in a way that they did not receive any chemotherapy or hormonal preoperatory treatment.

The tumor characteristics and number of metastatic lymphatic ganglia were collected originally from the ordinary pathology reports and samples were separated in two groups: the first group, consisting of 54 patients without metastasis (Table 1), named N0 (mean age 51.6 years old) and the second group, consisting of 64 samples of tumors with metastatic ganglia named N1 (mean age 51.75 years old).

The tumor samples, adjacent normal tissue, and ganglia were collected immediately after surgical extirpation of the tumor; tissues were fixed 48 h in 10% buffered formaldehyde solution and then embedded in paraffin at 56°C to blocks according to standard procedures of the pathology lab. Then embedded tumors were sectioned and samples stained with H&E. The samples were included in the study by their complete clinical history, as previously stated by inclusion criteria.

Microtome slices 3 *μ*m thick were taken from breast cancer paraffin blocks (microtome Reichert-Jung Mod 820-II), and slices were mounted in 26 × 76 mm polylysine treated (Sigma Chemical Co, EUA) laboratory glass (Deltalab). The slices were incubated at 56°C by 1 h to later remove the paraffin with xylol (Sigma Chem) and then proceed to gradual rehydration with 90, 80, and  70% ethanol as usual. Antigenic recovery treatment was performed as described in the provider's booklet; briefly, sodium citrate buffer solution was added to the samples (sodium citrate 10 mM and Tween 20 at 0.1%, pH = 8-9) and then warmed up to 116°C in a Pascal cooker (Dako Cytomation). Endogen peroxidases were inactivated with a 3% hydrogen peroxide solution in absolute methanol by 5 min and then rinsed up 4 min with wash buffer (1x PBS in 0.1% Tween 20). In order to block unspecific reactions, the sample is then incubated with 1% bovine serum albumin (BSA) (Sigma Chem) by 1 h. Recognizing of the specific antigen in turn is made by adding the IgG polyclonal/monoclonal antibody at the optimal concentration recommended by the provider and these reactions were incubated 30–60 min and then rinsed with the wash buffer above. Antibodies used in this study were the following: rabbit polyclonal anti-uPA (Genetex 0.4 mg/mL), rabbit polyclonal IgG anti-PAI-1 (Millipore 1 mg/mL), IgG anti-MMP-1 mouse monoclonal antibody (Santa Cruz 0.2 mg/mL), rabbit polyclonal anti-MMP-3 (Biovisión 0.5 mg/mL), rabbit polyclonal anti-MMP-9 (Genetex 0.5 mg/mL), rabbit polyclonal anti-TIMP-1 (0.5 mg/mL Biosystems), and rabbit monoclonal anti-uPAR (Genetex 0.5 mg/mL). Antigen-antibody reaction was detected by the Mouse/Rabbit PolyVue Plus™ HRP/DAB Detection System (Biosystem, California, USA) and then revealed as usual, by few minutes with chromogenic reactive diaminobenzidine tetrahydrochloride, DAB (Sigma Chem), and then rinsed with bidistilled water. Counterstaining of the immunolabeled samples was performed with Harris's hematoxylin by 45 sec. (Santa Cruz Biotechnology) and then treated with a lithium chloride saturated solution by 30 sec. Tissue preparations were mounted in appropriate resin and microscopy examined. Negative controls were included and prepared as the protocol established substituting the primary antibody by PBS solution containing 1% BSA. The controls for expression were placental tissue samples, which had overexpression of the molecule in turn as it is stated in the legends of each figure. Samples were microscopy visualized in 40x augmentation in an optical microscope (Olympus) with integrated photographic camera to obtain TIFF images in RGB in 24-bit color and 4164 × 3120 resolution. Images were processed and analyzed in Java background, using the software* ImageJ-v1.45p. *Background illumination was adjusted with the* Rolling Ball *algorithm to later recover the regions of interest (ROI).* Deconvolution* algorithm was used to exclusively separate the contribution of DAB from the background; then these images were analyzed to obtain the mean of optical density values (ODM). Our obtained values oscillated between 0 and 255 to finally yield the integral optical density (IOD) [21]. IOD represent the density or abundance of each developed signal per image; therefore it might safely represent the expression of each protein.

### 2.3. Statistical Analysis

The statistical analysis was performed with GraphPad Prism Software version 6 for Windows. The intensities of optical density (IOD) values for the analyzed proteins were compared using the nonparametric Mann–Whitney* U* test. Statistical significance was accepted when *p* < 0.05.

## 3. Results

**Figure 1:**
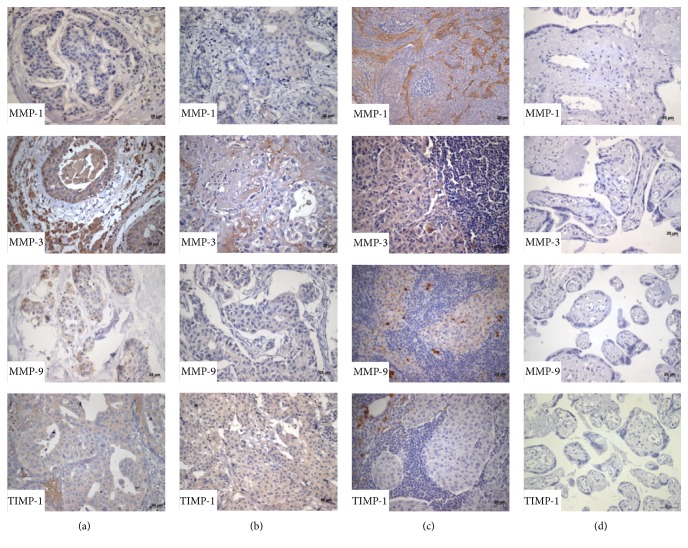
Expression of matrix metalloproteases in samples with ductal breast cancer and metastatic ganglia. Immune-chemistry with labeled matrix metalloproteinases MMP-1, MMP-3, MMP-9, and the metalloproteinase tissue specific inhibitor TIMP-1 in ductal breast cancer samples; (a) with metastasis, (b) without metastasis, (c) metastatic lymphatic ganglia diagnosed by trans-surgical H&E, and (d) placental tissues were negative controls. Magnification 400x.

**Figure 2:**
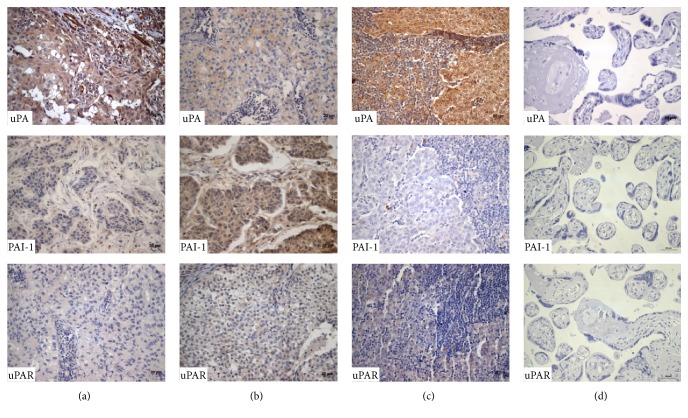
Expression of fibrinolytic system proteins in samples with ductal breast cancer. Representative images of the immunohistochemistry results of proteins from the fibrinolytic system uPA, uPAR, and PAI-1 in ductal breast cancer tissues with metastasis (a), without metastasis (b), metastatic ganglia (c), and negative controls (d). Magnification 400x.

Immunohistochemistry tests were performed in breast cancer sections in order to locate signal from each protein in the tissues, several members from the metalloproteinases system (MMP-1, MMP-3, MMP-9, and inhibitor TIMP-1) and also from the fibrinolytic system (uPA, uPAR, and PAI-1) were specifically screened, and then a comparison of their semiquantitative expression was obtained as a numeric value of signal intensity (IOD), in the two groups of cancer patients: N0 with no metastasis and N1 with metastatic cancer in close ganglia nodes. All the proteins showed to be actively expressed in both groups, directly in tumor cells (Figures 1 and 2). Besides, expression of this group of proteinases also was observed in the tumor surrounding cells, of several cell lineages, such as extracellular matrix cells, immune cells, fibroblasts, and endothelial cells (data not shown).

### 3.1. MMPs Had Differential Expression in Metastatic and No Metastatic Breast Cancer Samples

Evaluation of expression of MMP-1, MMP-3, MMP-9, and TIMP-1 in 54 samples from patients with no metastatic tumors in lymph nodes was indirectly estimated by obtaining numeric values of intensity IOD, from the corresponding immunohistochemistry results (standard process).

### 3.2. Results from No Metastatic Patients, Group (N0)

As it is shown in Table 2 where results from this section are resumed, the highest signal (IOD mean) belonged to MMP-3, in both metastatic and no metastatic tumor samples. MMP-3 was the most expressed MMP in ductal breast tumor cells (mean IOD value 22,300).

Regarding the levels of expression, MMP-3 signal is distantly followed by MMP-1 (mean IOD 15,500) and then MMP-9 (mean IOD 11,600), and finally the one with the lowest expression level was the metalloproteinases inhibitor, TIMP-1 (mean IOD 10,600). Nevertheless, differential expression of metalloproteases was established in this study, since MMPs from the metastatic group (N1) showed differences in intensities comparing to the N0 group without metastasis.

### 3.3. Results from the Metastatic Patients Group (N1)

As from the 64 tumors with at least one metastatic ganglia, results showed that MMP-3, MMP-1, MMP-9, and TIMP-1 kept the same order of intensity present in N0; nevertheless, N1 showed higher intensity mean values. The highest signal was from MMP-3 like it does in the metastatic tumors (mean IOD 28,600), followed by MMP-1 (mean IOD 20,200), MMP-9 (mean IOD 13,200), and TIMP-1 (mean IOD 12,300). Expression of all studied MMPs can be easily compared in Table 2. Interestingly, when comparing the intensities detected for MMP-3 in N1 tumors (28,600), it was higher than the one from the no metastatic tumors N0 (22, 300).

### 3.4. Fibrinolytic System Expression in No Metastatic Breast Cancer, Group N0

As well as above, expression of uPA, uPAR, and PAI-1 was screened in 54 samples of breast cancer with no metastatic ganglia, followed by estimation of the intensity IOD. Results from the group without metastasis N0 showed the highest level of expression from uPA, directly in tumor cells (mean IOD 26,000), followed by PAI-1 (mean IOD 25,100) and uPAR (mean IOD 19,800).

### 3.5. Detection of Proteins from the Fibrinolytic System in Metastatic Breast Cancer, Group N1

Evaluation of expression from uPA, uPAR, and PAI-1 from 64 samples of patients with close ganglia metastasis (N1) was obtained as described above and the results can be easily compared in Table 2. Again uPA showed the highest values of expression in tumor cells (mean IOD 37,600), followed by PAI-1 (mean IOD 19,200) and uPAR (mean IOD 20,000).

### 3.6. Comparison between N0 and N1 Groups

**Figure 3:**
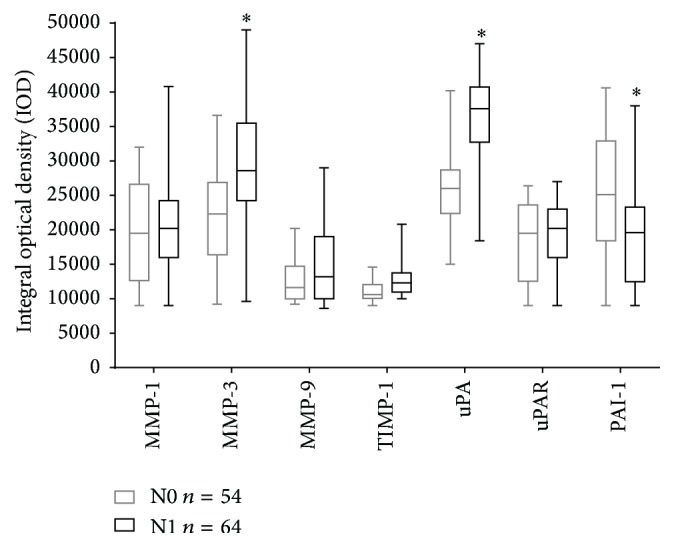
Semiquantitative expression of MMPs and the fibrinolytic system proteins in breast cancer samples. Expression of MMPs (MMP-1, MMP-3, MMP-9, and TIMP-1) and proteins from the fibrinolytic system (uPA, uPAR, and PAI-1) in ductal breast cancer samples with metastasis (N1) and without metastasis (N0). Semiquantitation of the signal was performed using ImageJ software and statistic significance acquired applying the Mann–Whitney* U* test ^*∗*^ *p* < 0.05.

When semiquantitative expression was compared among N0 and N1 (Figure 3), the no metastatic and metastatic patient groups, respectively, intensity from MMP-3 was 28% higher in the metastatic group (N1) comparing to the no metastatic group (N0); besides uPA was 45% higher in the metastatic (N1) than in the no metastatic group (N0).

As it is easily noted in Table 2, higher expression of MMP-3 in metastatic samples correlates also with histologic grades GI and GII but none with GIII, also corresponding to the size tumor T2 and the tumor phenotype (*Luminal* A) based in the observation of metastatic ganglia (*p* < 0.05). More importantly, elevated uPA showed a direct correlation with metastatic tumors independently of histologic grade, size, or tumor phenotype (*p* < 0.05). Furthermore, regarding the tumor grade, semiquantitation of PAI-1 (direct inhibitor of uPA) was 24% lower in tumors with metastatic ganglia (N1) in comparison with the group without metastasis (N0). Besides, a decrement expression of PAI-1 statistically correlates with Grade I and II tumors, with T2 size having either phenotype A or phenotype B.

In our hands, there were no statistically significant differences of signal levels from MMP-1, MMP-9, uPAR, or TIMP-1. Instead, a tendency to diminish the expression of MMP-9 in no metastatic tumors (N0) was observed (Figure 1(b)). Interestingly, the samples with metastasis in this study showed a tendency to an increased expression of TIMP-1 in comparison with samples from patients without metastasis. Those results must be considered a tendency, since they had not statistically significant support; anyway this fact is mentioned, because other authors [22, 23] previously reported elevated TIMP-1 in association with metastasis, suggesting that the tendency observed in our studies is perhaps real (and statistics could be easily corrected increasing the number of samples). In the interim, we have not enough evidence to support this molecule as a participant in the occurrence of metastasis in ductal breast cancer.

### 3.7. Detection of MMPs and Fibrinolytic System Members in Metastatic Lymphatic Nodes

Several lymphatic ganglia nodes with metastasis (64) were available for immunohistochemistry; detection of the same markers was performed: MMP-1, MMP-3, MMP-9, TIMP-1, uPA, uPAR, and PAI-1. The results showed that not only was signal from these proteases present in the cytoplasm of neoplastic cells, but as well it was present in the immune mononuclear infiltrates, and even the tumor extracellular matrix (Figure 1(c)).

Semiquantitation of the markers was performed in 64 metastatic lymphatic ganglia from ductal breast cancer patients, followed by the usual IOD estimation. Expression of these proteins in neoplastic cells located in the ganglia was quantitated and resulted as follows, MMP-3 (mean IOD 22,200), MMP-9 (mean IOD 20,900), MMP-1 (mean IOD 16,600), TIMP-1 (mean IOD 17,800), uPA (mean IOD 39,400), uPAR (mean IOD 21,800), and PAI-1 (mean IOD 18,000).

The results obtained from cancer cells at the lymphatic ganglia were considerably higher than the ones from the metastatic tumors N1 and further higher from no metastatic tumors in N0 (Table 2). Additionally, after performing a comparison of expression of each marker in the metastatic cells, against the primary tumor cells, the IOD means confirmed an increment of expression. Particularly a 58% increment of MMP-9 expression was exhibited in neoplastic cells in the lymphatic ganglia. The opposite occurred when the comparison was performed for MMP-3, showing a 28% diminished expression in lymphatic ganglia. In addition, a not statistically significant but rather a numeric tendency of diminished expression in metastatic ganglia is apparent when MMP-1 and PAI-1 were analyzed by comparison with the expression of the primary tumor cells (Table 2).

### 3.8. Phenotypic Switch on Mexican Women

Perhaps it is important to point out that from the total of breast cancer Mexican patients' with and without metastasis analyzed in this study (Table 1), an apparent switch in the Luminal phenotype is shown.

A 40% of the 118 patients showed phenotype* Luminal B* (RE+, RP+/−, HER2+). This would be a contrasting result compared to those from several authors, that studying white populations had found predominantly the* Luminal A* phenotype [24].

We have seen reports from the literature where the predominant phenotype is* Luminal A* (RE+, RP+/−, and HER2−) in up to 60% of patients [25]. Apparently the results from this study make sense, since there are also authors mentioning an apparent switch in Luminal phenotype, due to breast cancer patients of Hispanic origin having a major tendency to be Her2+, in comparison with no Hispanic white population. Data from those authors [25] are supportive to the present finding of the majority of* Luminal B *and* Her2+* phenotype in our sample. In this study the patients other than* Luminal B* (60%) can be sorted in three phenotypes: the* Luminal* A, the Her2+ phenotype, and the triple negative phenotype (Table 1). It is important to mention that in the N1 group of metastatic patients, the percentage of* Luminal B* phenotype (RE+, RP+/−, and HER2+) was as high as 45.5% (Table 1), which is considerably higher than the media value of 40% calculated for the total of the samples; it is indeed a considerably higher percentage when we consider that for the N0 group of no metastatic patients, the* Luminal* B phenotype ranged in 31.5%, what seems to point out that* Luminal B* phenotype may be somehow related to metastatic tumors, although the significance of this observation still would not be sufficiently supported by only one study.

## 4. Discussion

Crucial events in breast cancer tumor invasion and further metastasis are degradation of the basal membrane, extracellular matrix, and access to blood vessels [26]. Not only the tumor cells, but also other resident cells of the tissue harboring metastasis, plus the inflammatory cells, are also capable of supplying proteolytic enzymes that may facilitate metastasis, such as MMPs and the fibrinolytic system proteins. These proteases are able to process different kinds of collagen and other proteins of ECM, aiding with these actions the processes of tumor invasion and metastasis [27]. Overexpression of these proteinases was already associated with tumor progression and survival rate of cancer patients [28, 29]. Prognostic and predictive factors, such as tumor size, tumor grade, tumor phenotype, or ganglia metastasis, all have a key role in the correct diagnoses and treatment of breast cancer patients. All these factors are taken together, screened, and evaluated in order to excel in establishing an accurate and prompt diagnose, so the patient life is protected by granting the best possibility of receiving a well-timed, appropriate, and effective treatment.

The main purpose of this study is to consider the expression of several members of the ECM remodeling metalloproteinases and fibrinolytic systems, due to their primary intervention in generation of metastasis, and possibly correlate the results, so patients might get benefits of an early detection of metastasis. We believe that if a correlation is established between expression levels of some of these proteinases, with histologically diagnosed metastatic ganglia, detection of this proteinase in histopathology samples may increase the certainty of the prognosis of metastasis. The use of detecting overexpression of one or more of the studied markers may perhaps allow an early diagnosis of metastasis, even in the absence of metastatic ganglia in the trans-surgery histopathology. We hope that the earliest diagnosis of metastasis would be the most appealing prognostic factor that would be considered to foresee a clinical evolution of metastasis in breast cancer.

Regarding the molecular studies of metalloproteases and fibrinolytic system in relation to metastasis, overexpression of MMP-3 and uPA associated with metastasis of lymphatic ganglia has been previously found, perhaps this is the kind of activity that could be enhancing metastasis towards different organs [30].

The above findings make more sense after the observation that exactly the opposite activity (overexpression of PAI-1) was taking place in tumors rather than in ganglia, of the no metastatic lesions group (N0); this fact opens the opportunity to suggest that PAI-1, perhaps through the regulation of its counterpart uPA, might be an important event for metastasis to take place. In other words, PAI-1 in no metastatic tumors (N0) directly regulates the activation of uPA and, therefore, probably when uPA is out of control metastasis would start.

Even though our results suggest an antimetastatic role for uPA, there are authors proposing exactly the opposite, that uPA might be a prometastatic molecule, since it is overexpressed in high grade tumors already with metastasis in lymphatic nodes [31–33]; the difference is that they did not make a comparison with tumors lacking metastatic ganglia, as we actually did.

In our hands, overexpression of MMP-3 and uPA in tumor cells, and perhaps with a further contribution from the mononuclear infiltrate and stroma, would be directly related to metastasis in ganglia.

Moreover, an elevated expression of MMP-3 and uPA together appears to increase, altogether with the tumor grade and size; therefore our results are more alike to those in the literature, showing direct correlation between expression of uPA with high grade of the tumor, size, and lymphovascular invasion [31–33].

Furthermore, another way of promoting metastasis has been already suggested, and it is based on an effective induction of angiogenesis, in order to better irrigate the tumor. In this study the tumors with metastasis in ganglia have increased levels MMP-3 and uPA; thus the possibility exist for these proteases to be also promotors of formation of new blood vessels, and according also to other authors [34–36] the mentioned overexpression of these proteinases might be in fact involved with regulation of the process of angiogenesis, but perhaps indirectly, at the same time they are also favoring metastasis.

Additionally, since metalloproteases are regulated by feedback processes altogether with its corresponding inhibitors, such activity might be directly depending on specific inhibitors such as TIMP-1.

While studying the statistical significance of our evidence pointing out TIMP-1 as a participant in the process of metastasis, in this comparative study TIMP-1 has shown a tendency, involving its presence in metastatic tumors. Since other colleagues studied this molecule and they found TIMP-1 associated with metastasis, we always had in mind that this molecule may have a role in metastasis but we were simply unable to support that fact with the size of our studied sample. Perhaps a greater sample would show significance of TIMP-1 and its involvement with metastasis.

Since metastasis in cancer progression is the result of multiple interactions among tumor cells and the tumor microenvironment, a fine understanding of these relationships may allow a better comprehension of the tumor biology. Achieving little more knowledge of tumor biology is one of the goals of this particular study. Because of that, the authors agreed to address the importance of the molecular study of the remodeling proteins in the light of metastasis progression. We also agreed that a good start in this path would be the search for MMPs and the fibrinolytic systems in metastatic and no metastatic tumor sections of ductal breast cancer, since the most frequent tumors in the Hospital Juárez de México were those, so we carried out the study in the hope that somehow this molecular characterization would be directly useful to Mexican patients undergoing this cancer.

Although our study approaches molecules from these systems expressed exclusively by tumor cells, tumor micro environmental cells expressing them were also photo-documented (data not shown), considering that all collected data might somehow become a guide towards new strategies of antitumor pharmacotherapy, particularly those focused in abolishing the generation of metastasis in breast cancer patients. Perhaps designing of more successful therapies would be possible by targeting the tumor cells and the micro-environmental cells, particularly the ones from the immune system.

The experimental strategy designed for this approach has been the selection of samples from patients with the same type of cancer: ductal breast cancer tumors, with or without metastasis in the first approachable ganglia such as axillar, in the assumption that patients would have comparable lesions, and that the difference among the two groups would be only metastasis in ganglia. To further document the results from the tumor samples, the study was extended to explore the same molecular markers in the cancer lesions of the ganglia.

Collected data from the phenotype in samples from Mexican patients in this study resulted to be very similar to reports from other authors working with populations other than Hispanic origin patients, pointing out an apparent phenotypic switch from breast cancer* Luminal A *more common in the white population, to* Luminal* B, predominant in Hispanic patients. In fact,* Luminal B* is the predominant phenotype in samples of this study, as it occurs in the Hispanic populations.

It is important to us to mention that in the N1 group of metastatic patients,* Luminal B* phenotype (RE+, RP+/−, and HER2+) was shown to be 45.5% higher than the media of 40% calculated for the total of the samples. The group of patients without metastasis only had 31.5% of* Luminal B* phenotype what seems to suggest that* Luminal B* is somehow related to metastasis. Since the significance of this observation is still not well discussed in the literature, we just open the discussion to our colleagues. The above results were observed in a sample of Mexican women, attending a public facility: Hospital Juárez de México in Mexico City. These results can be matched with Hispanic populations or Mexicans living all over the world.

## 5. Conclusion

Our data strongly suggest that activity of both systems, MMPs and fibrinolytic, responsible of remodeling of tissues in no pathologic conditions, actually becomes key factor in tumor metastasis and perhaps also tumor progression (although this parameter was not directly addressed in this study), due to their participation in the processing of macromolecules of the stroma and in general providing favorable conditions for tumor metastasis. In this study we were able to distinguish a statistically significant increase of MMP-3 and uPA produced by the tumor cells in breast cancer tumor samples. Although the production of these molecules was not restricted to cancer cells, since they also were observed to be produced by other stripes from the tumor microenvironment, in the end they might be important for metastatic progression, perhaps facilitating migration of metastatic tumor cells. The importance of these systems in the invasion process was not addressed here.

Besides, since the fibrinolytic system enzyme uPA directly depends on its inhibitor PAI-1 for specific feedback regulation and metastatic tumors showed reduced levels of PAI-1, we suggest that this particular system may have a role in metastasis promotion. Additional experimental data suggested that tumor microenvironmental cells like endothelial cells, stromal cells, and also cells from the immune response express also molecules from the fibrinolytic and metalloprotease systems. Possibly tumor environmental cells are modulated by cancer cells to enhance the expression of fibrinolytic and metalloprotease enzymes.

Results from this study strongly suggest that the increased expression of MMP-3 and uPA at the same time that a PAI-1 decrement in the same tissues might be an important step for development of metastasis towards lymphatic ganglia, as shown in Mexican breast cancer patients. Nevertheless, this group of molecules should be studied in similar breast cancer lesions from other populations, to collect more experimental evidence, perhaps supporting the use of these molecules as prognostic factors of metastasis. Additionally, follow-up studies of such patients would shed more light on establishing a greater probability of development of metastasis, the kind of data that may be helping decisions of prompt surgical intervention and then appropriate supplemental therapy and the appropriate patient follow-up.

Finally, we conclude that results from this experimental comparative study of metastatic and no metastatic tumor samples, strongly support a statistically significant elevation of expression for uPA and MMP-3 molecules, in primary tumors of metastatic breast cancer, compared to its counterparts without metastasis.

## Figures and Tables

**Table 1:** Clinical and pathological characterization of breast cancer patients participating in the study.

Clinical-pathological characteristics	All cases (118) Ductal breast cancer	%	Groups according to metastasis			
			N0		N1	
			Cases (54)	%	Cases (64)	%
Age (years)						
≤55	77	65	32	60	45	70
>55	41	35	22	40	19	30
Tumor size						
T1 (<2 cm)	25	21	13	24	12	19
T2 (2–5 cm)	79	67	35	65	44	69
T3 (>5 cm)	14	12	6	11	8	12
Tumor grade (SBR)						
I	29	25	17	31	12	19
II	65	55	29	54	36	56
III	24	20	8	15	16	25
Nodal status						
pN0	54	46	54	100		
pN1	30	25			30	47
pN2	28	24			28	44
pN3	6	5			6	9
Mononuclear infiltrate						
Positive	85	72	39	72	46	72
Negative	33	28	15	28	18	28
Desmoplasia						
Positive	42	36	19	35	23	36
Negative	76	64	35	65	41	64
Molecular subtype						
Luminal A	29	25	12	22	17	27
Luminal B	46	39	17	31	29	45
Her2	25	21	16	30	9	14
Triple negative	18	24	9	17	9	14

Patients (*n* = 118), with invasive ductal breast cancer, were sorted in two groups based on presence/absence of metastasis in nearest ganglia. N0 = without metastasis in ganglia nodes. N1 = with at least one metastatic ganglia node.

**Table 2:** Semiquantitation of protein samples analyzed in patients with ductal breast cancer.

Protein	Group			*p* value	
	N0 IOD (mean)	N1 IOD (mean)	Nodal metastatic IOD (mean)		
				N0 versus N1	N1 versus nodal
MMP-1	15,500	20,200	16,600		
MMP-3	22,300	28,600	22,200	<0.05	<0.05
MMP-9	11,600	13,200	20,900		<0.05
TIMP-1	10,600	12,300	17,800		
UPA	26,000	37,600	39,400	<0.05	
UPAR	19,800	20,000	21,800		
PAI-1	25,100	19,200	18,000		

Semiquantitation of the signal was performed using ImageJ software and statistical significance acquired by applying the Mann–Whitney *U* test *p* < 0.05. N0 = without metastasis in ganglia nodes. N1 = with at least one metastatic ganglia node.

## Abbreviations

ECM: Extracellular matrix
H&E: Hematoxylin and eosin
MMPs: Matrix metalloproteinases
PAI-1: Plasminogen activator inhibitor 1
uPA: Urokinase-type plasminogen activator
TIMP-1: Tissue inhibitor of metalloproteinases 1.

## References

[1] Torre L. A., Bray F., Siegel R. L., Ferlay J., Lortet-Tieulent J., Jemal A. (2015). Global cancer statistics, 2012. *CA: A Cancer Journal for Clinicians*.

[2] Geiger T. R., Peeper D. S. (2009). Metastasis mechanisms. *Biochimica et Biophysica Acta—Reviews on Cancer*.

[3] Bacac M., Stamenkovic I. (2008). Metastatic cancer cell. *Annual Review of Pathology: Mechanisms of Disease*.

[4] Gupta G. P., Massagué J. (2006). Cancer metastasis: building a framework. *Cell*.

[5] Scully O. J., Bay B.-H., Yip G., Yu Y. (2012). Breast cancer metastasis. *Cancer Genomics and Proteomics*.

[6] Hadler-Olsen E., Winberg J.-O., Uhlin-Hansen L. (2013). Matrix metalloproteinases in cancer: their value as diagnostic and prognostic markers and therapeutic targets. *Tumor Biology*.

[7] Deryugina E. I., Quigley J. P. (2006). Matrix metalloproteinases and tumor metastasis. *Cancer and Metastasis Reviews*.

[8] Chaffer C. L., Weinberg R. A. (2011). A perspective on cancer cell metastasis. *Science*.

[9] Smith H. A., Kang Y. (2013). The metastasis-promoting roles of tumor-associated immune cells. *Journal of Molecular Medicine*.

[10] López-Otín C., Matrisian L. M. (2007). Emerging roles of proteases in tumour suppression. *Nature Reviews Cancer*.

[11] Noël A., Jost M., Maquoi E. (2008). Matrix metalloproteinases at cancer tumor-host interface. *Seminars in Cell and Developmental Biology*.

[12] Gill S. E., Parks W. C. (2008). Metalloproteinases and their inhibitors: regulators of wound healing. *International Journal of Biochemistry and Cell Biology*.

[13] Malemud C. J. (2006). Matrix metalloproteinases (MMPs) in health and disease: an overview. *Frontiers in Bioscience*.

[14] Park H. I., Ni J., Gerkema F. E., Liu D., Belozerov V. E., Sang Q.-X. A. (2000). Identification and characterization of human endometase (matrix metalloproteinase-26) from endometrial tumor. *The Journal of Biological Chemistry*.

[15] Khokha R., Murthy A., Weiss A. (2013). Metalloproteinases and their natural inhibitors in inflammation and immunity. *Nature Reviews Immunology*.

[16] Kwaan H. C., Mcmahon B. (2009). The role of plasminogen-plasmin system in cancer. *Cancer Treatment and Research*.

[17] Crippa M. P. (2007). Urokinase-type plasminogen activator. *International Journal of Biochemistry and Cell Biology*.

[18] Cesarman-Maus G., Hajjar K. A. (2005). Molecular mechanisms of fibrinolysis. *British Journal of Haematology*.

[19] García M. F., González-Reyes S., González L. O. (2010). Comparative study of the expression of metalloproteases and their inhibitors in different localizations within primary tumours and in metastatic lymph nodes of breast cancer. *International Journal of Experimental Pathology*.

[20] Malinowsky K., Wolff C., Berg D. (2012). uPA and PAI-1-related signaling pathways differ between primary breast cancers and lymph node metastases. *Translational Oncology*.

[21] Ruifrok A. C., Johnston D. A. (2001). Quantification of histochemical staining by color deconvolution. *Analytical and Quantitative Cytology and Histology*.

[22] Zhang M., Teng X.-D., Guo X.-X., Li Z.-G., Han J.-G., Yao L. (2013). Expression of tissue levels of matrix metalloproteinases and their inhibitors in breast cancer. *Breast*.

[23] Dechaphunkul A., Phukaoloun M., Kanjanapradit K. (2012). Prognostic significance of tissue inhibitor of metalloproteinase-1 in breast cancer. *International Journal of Breast Cancer*.

[24] Dawood S., Hu R., Homes M. D. (2011). Defining breast cancer prognosis based on molecular phenotypes: Results from a Large Cohort Study. *Breast Cancer Research and Treatment*.

[25] Hines L. M., Risendal B., Byers T., Mengshol S., Lowery J., Singh M. (2011). Ethnic disparities in breast tumor phenotypic subtypes in Hispanic and non-Hispanic white women. *Journal of Women's Health*.

[26] Bourboulia D., Stetler-Stevenson W. G. (2010). Matrix metalloproteinases (MMPs) and tissue inhibitors of metalloproteinases (TIMPs): positive and negative regulators in tumor cell adhesion. *Seminars in Cancer Biology*.

[27] Ham M., Moon A. (2013). Inflammatory and microenvironmental factors involved in breast cancer progression. *Archives of Pharmacal Research*.

[28] Duffy M. J., Maguire T. M., Mcdermott E. W., O'Higgins N. (1999). Urokinase plasminogen activator: a prognostic marker in multiple types of cancer. *Journal of Surgical Oncology*.

[29] Jezierska A., Motyl T. (2009). Matrix metalloproteinase-2 involvement in breast cancer progression: a mini-review. *Medical Science Monitor*.

[30] Liu D., Guo H., Li Y., Xu X., Yang K., Bai Y. (2012). Association between polymorphisms in the promoter regions of matrix metalloproteinases (MMPs) and risk of cancer metastasis: a meta-analysis. *PLoS ONE*.

[31] Andres S. A., Edwards A. B., Wittliff J. L. (2012). Expression of urokinase-type plasminogen activator (uPA), its receptor (uPAR), and inhibitor (PAI-1) in human breast carcinomas and their clinical relevance. *Journal of Clinical Laboratory Analysis*.

[32] Hildenbrand R., Schaaf A. (2009). The urokinase-system in tumor tissue stroma of the breast and breast cancer cell invasion. *International Journal of Oncology*.

[33] Schmitt M., Mengele K., Gkazepis A. (2008). Assessment of urokinase-type plasminogen activator and its inhibitor PAI-1 in breast cancer tissue: historical aspects and future prospects. *Breast Care*.

[34] Rakic J. M., Maillard C., Jost M. (2003). Role of plasminogen activator-plasmin system in tumor angiogenesis. *Cellular and Molecular Life Sciences*.

[35] Pepper M. S. (2001). Role of the matrix metalloproteinase and plasminogen activator-plasmin systems in angiogenesis. *Arteriosclerosis, Thrombosis, and Vascular Biology*.

[36] Jackson C. (2002). Matrix metalloproteinases and angiogenesis. *Current Opinion in Nephrology and Hypertension*.

